# Blood cell-derived extracellular vesicles: diagnostic biomarkers and smart delivery systems

**DOI:** 10.1080/21655979.2021.1982320

**Published:** 2021-10-08

**Authors:** Limei Xu, Yujie Liang, Xiao Xu, Jiang Xia, Caining Wen, Peng Zhang, Li Duan

**Affiliations:** a Department of Orthopedics, The First Affiliated Hospital of Shenzhen University, Shenzhen Second People’s Hospital, Shenzhen, Guangdong, China; b Guangdong Provincial Research Center for Artificial Intelligence and Digital Orthopedic Technology, Shenzhen Second People’s Hospital, Shenzhen, Guangdong, China; c Shenzhen Institute of Advanced Technology, Chinese Academy of Sciences, Shenzhen, Guangdong, China; d Shenzhen Kangning Hospital, Shenzhen Mental Health Center, Shenzhen, Guangdong, China; e Department of Chemistry, and Center for Cell & Developmental Biology, School of Life Sciences, the Chinese University of Hong Kong, Shatin, Hong Kong SAR, China; f Shenzhen Institute of Geriatrics, Shenzhen, Guangdong Province, China

**Keywords:** Blood cells, extracellular vesicles, biomarkers, targeted delivery

## Abstract

Extracellular vesicles (EVs) are released by most of the cells or tissues and act as nanocarriers to transfer nucleic acids, proteins, and lipids. The blood system is the most abundant source of extracellular vesicles for purification, and it has attracted considerable attention as a source of diagnostic biomarkers. Blood-derived extracellular vesicles, especially vesicles released from erythrocytes and platelets, are highly important in nanoplatform-based therapeutic interventions as potentially ideal drug delivery vehicles. We reviewed the latest research progress on the paracrine effects and biological functions of extracellular vesicles derived from erythrocytes, leukocytes, platelets, and plasma. From a clinical perspective, we summarize selected useful diagnostic biomarkers for therapeutic intervention and diagnosis. Especially, we describe and discuss the potential application of erythrocyte-derived extracellular vesicles as a new nano-delivery platform for the desired therapeutics. We suggest that blood-derived extracellular vesicles are an ideal nanoplatform for disease diagnosis and therapy.

## 1. Introduction

Extracellular vesicles (EVs) are nanoscale lipid bilayer particles released by various cell types. Studies have demonstrated that extracellular vesicles can selectively carry biological cargoes, such as nucleic acids, proteins, and lipids, from their maternal cells, importantly, extracellular vesicles can deliver their biological cargoes and act as messengers in intracellular communication [1,2]. Therefore, extracellular vesicles are extensively involved in physiological regulation and pathological processes [3].

Extracellular vesicles are distributed in almost all body fluids, such as peripheral blood, sweat, saliva, and urine [4]. Peripheral blood is routinely adopted for clinical diagnosis since it is conveniently available for sampling and testing. Recently, peripheral blood vesicles have attracted considerable attention both as drug delivery vehicles and as biomarkers for disease diagnosis and prognosis [5–8]. However, peripheral blood vesicles are a mixture released by different cell types, including erythrocytes, leukocytes, and platelets. Extracellular vesicles carriers originating from specific cell types rather than complete peripheral blood, which may significantly improve their drug delivery efficiency, were developed. Extracellular vesicles derived from erythrocytes lack nuclear and mitochondrial DNA and do not result in gene transfer [9], which are promising therapeutic drug carriers. This review describes the biological functions of extracellular vesicles released from erythrocytes, leukocytes, platelets and plasma and the roles of these extracellular vesicles in therapeutic intervention and drug delivery. We also discuss some exciting results of our recent study on erythrocyte-derived extracellular vesicles (REVs) as drug delivery carriers, which may provide strong evidence for the development of excellent drug delivery carriers for therapeutic use.

## 2. Extracellular vesicles derived from erythrocytes

**Figure 1.**
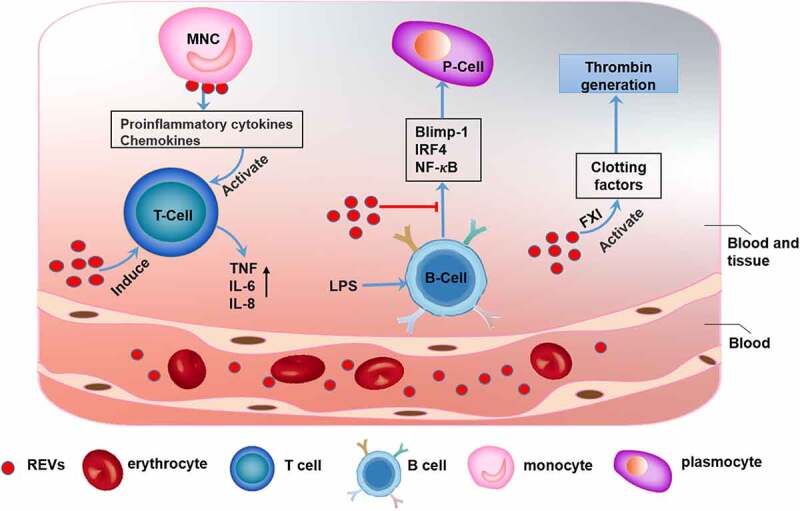
**The function of erythrocyte-derived extracellular vesicles** (**REVs)** REVs elicit immune-inflammatory responses by modulating the biological activities of both T cells and B cells. REVs stimulate monocytes to produce proinflammatory cytokines and chemokines, which promote T cell proliferation and further stimulate T cells to produce TNF, IL-6, and IL-8. REVs also inhibit the expression of Blimp-1 and IRF4 and activation of the NF-κB pathway, which inhibit B cell function. Additionally, REVs mediate blood coagulation by activating coagulation factors such as FXI, which initiates and promotes thrombin production

Erythrocytes are the most abundant cell type in peripheral blood [9]. REVs participate in various physiological and pathological activities (Figure 1). REVs have essential physiological functions in blood clotting. Data have shown that REVs have coagulation factor XI (FXI)-dependent procoagulant properties and can activate clotting factors and initiate and propagate thrombin generation [10]. Adverse transfusion reactions may occur during the transfusion of erythrocytes. Studies have shown that transfusion-related immune regulation (TRIM) is associated with increased infection rates, reduced cancer survival rates, and short-term death after blood transfusion [11–13]. EVs derived from erythrocytes subjected to prolonged storage are closely related to the immune and inflammatory responses to blood transfusion. Further studies showed that REVs can also bind to monocytes and induce the release of proinflammatory cytokines, e.g., interleukin 1 (IL-1), interleukin 6 (IL-6), tumor necrosis factor-α (TNF-α) and chemokines, e.g., macrophage-derived chemokine (MDC) and macrophage inflammatory protein 1a (MIP-1a), which boost mitogen-driven T cell proliferative responses [14]. In addition, REVs can inhibit the expression of transcription factors, e.g., B lymphocyte-induced mature protein 1 (Blimp-1) and interferon regulatory factor 4 (IRF4) and activation of the NF-κB pathway in lipopolysaccharide (LPS)-primed B cells [15], which inhibits the B cell-mediated immune response. However, REVs also induce strong host responses with production of TNF, IL-6, and IL-8 [16]. Therefore, REVs are closely associated with adverse effects of blood transfusion [17,18]. In addition, REVs are involved in pathological processes, including Parkinson’s disease. Studies have found that the α-synuclein (α-syn) content in REVs is elevated in patients with Parkinson’s disease. REVs can carry α-syn and accumulate in astrocyte end-feet, which are a component of the blood-brain barrier (BBB). REV accumulation impairs the interaction between excitatory amino acid transporter 2 (EAAT2) and oligomeric α-syn, thus inhibiting glutamate uptake by astrocytes. This decrease in glutamate uptake can initiate and promote the progression of Parkinson’s disease [19] (Table 1).

**Table 1.** Significance of blood-derived EV-related molecules in diseases

EV type	Related molecules	Functional change	Possible mechanism	Disease	Ref.
REVs	α-syn	elevated	excess oligomeric a-syn interacts with EAAT2 to inhibit glutamate uptake by astrocytes	Parkinson’s disease	19
LEVs	miR-146a, miR-128, miR-185, miR-365, and miR-503	elevated	these miRNAs decrease cell migration and promote macrophage entrapment in the vessel wall	atherosclerosis	43
PEVs	serotonin	elevated	platelet-derived serotonin promotes the transit of PEVs to lymph nodes, activates autoantibodies, and increases vascular permeability	rheumatoid arthritis	57
plasma EVs	transforming growth factor-beta 1 (TGF-β1)	elevated	TGF-β1 significantly inhibits NK cell activity in cytotoxicity assays	relapsed leukemia	67
plasma EVs	miR-212 and miR-132	decreased	these miRNAs lose their protective effect on neurons	Alzheimer’s disease	68
plasma EVs	α-syn	elevated	α-syn activates microglia and astroglia, enhancing neurodegeneration	Parkinson’s disease	69

Extracellular vesicles, especially small vesicles have been developed as drug delivery carriers due to their excellent compatibility and ability to cross various biological barriers, such as the BBB. Moreover, compared to viruses, lipid nanomaterials, and lipid transfection agents, EVs generlly show low immunogenicity and cytotoxicity [20–23] and have great application potential in drug delivery [24–27]. REVs can deliver antisense oligonucleotides (ASOs) and suppress breast cancer progression in mice bearing breast cancer xenografts, without inducing systemic inflammation or toxicity to the liver or other organs [28]. Additionally, REVs loaded with antimalarial drugs (e.g., atovaquone and tafenoquine) can significantly inhibit the growth of Plasmodium falciparum in vitro [29] (Table 2).

**Table 2.** Summary of blood cell-derived EVs for drug delivery

EV source	Cargo	Loading method	Disease	Ref.
erythrocytes	antisense oligonucleotides	electroporation	breast cancer	28
erythrocytes	atovaquone and tafenoquine	coincubation	P. falciparum	29
platelets	TPCA-1	coincubation	pneumonia	59
plasma	miR-31 and miR-451a	electroporation	liver cancer	75

*TPCA-1*, [5-(p-fluorophenyl)-2-ureido] thiophene-3-carboxamide

**Figure 2.**
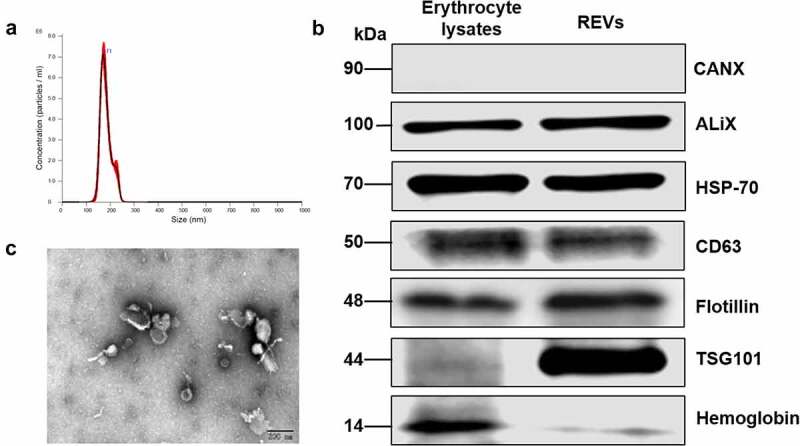
Characterization of erythrocyte-derived extracellular vesicles (REVs)

The characteristics of REVs, which do not carry a potential risk of gene transfer and are easily obtainable, have attracted considerable attention for nanocarrier development. We isolated peripheral blood erythrocytes from rats and treated the cells overnight with calcium ionophore, REVs were precipitated using ultracentrifugation. We then identified the biological characteristics of the obtained REVs. The nanoparticle tracking analysis (NTA) results suggested that the particle size was approximately 100–200 nm (Figure 2a). The RBC protein hemoglobin and EV markers (ALIX, HSP-70, CD63, Flotillin, and TSG101) were enriched in REVs (Figure 2b). The typical tea tray-like membrane structure of small extracellular vesicles was visualized by transmission electron microscopy (TEM) (Figure 2c). Interestingly, we identified tail-like structures using TEM, but the exact nature and function of these tail-like structures are not known.

The typical morphology of REVs was characterized using TEM and NTA. The biomarkers of REVs were detected using Western blotting.(A) Size distribution of REVs analyzed using NTA.(B) Western blot analysis of CANX, ALIX, HSP-70, CD63, Flotillin, TSG101, and hemoglobin in erythrocyte lysates and REVs.(C) TEM images of REVs. Scale bar, 200 nm.

Peripheral blood erythrocytes are the most abundant cell type in the blood [30]. Erythrocytes are easy to obtain, and their safety has been verified during many years of routine blood transfusion. Most importantly, since erythrocytes lack nuclear DNA and mitochondrial DNA [9], REVs will not cause gene transfer, and may be used as a good nanodrug delivery carriers. However, natural REVs have low targeting ability. In our future studies, we will focus on improving the function of REVs as nanocarriers via chemical modification.

## 3. Extracellular vesicles derived from leukocytes

**Figure 3.**
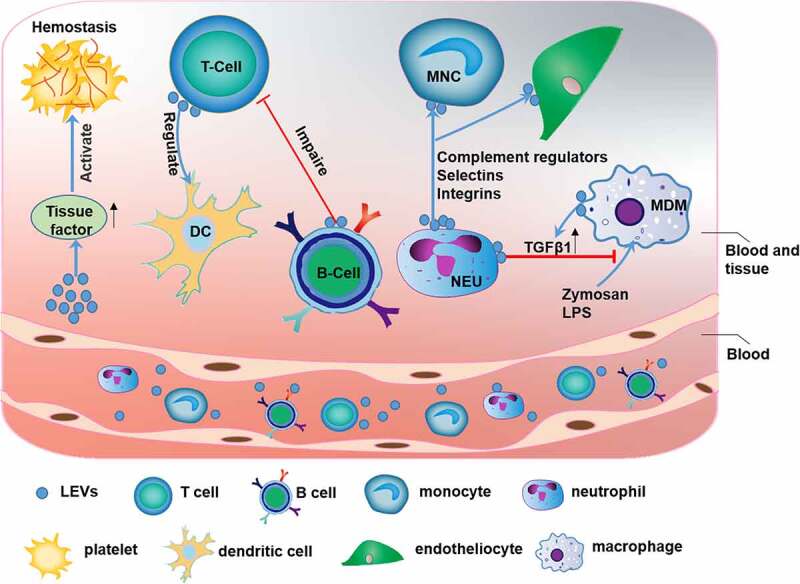
The function of leukocyte-derived extracellular vesicles (LEVs)

Leukocytes can be classified as granulocytes, lymphocytes, and monocytes based on their morphological characteristics. The primary function of leukocyte-derived EVs (LEVs) is to induce the immune response that is responsible for recognizing and removing pathogenic or harmful substances [31]. LEVs also participate in immune, and inflammatory responses and coagulation functions (Figure 3). A previous study reported that LEVs may be used as biomarkers for inflammatory and immunological disorders [32]. EVs released by granulocytes express a subset of cell surface proteins, *e.g*., selectins, integrins, and complement regulators, and activate the classical complement pathway in inflammation and cell signaling by adhering specifically to monocytes and endothelial cells [33] [34].However, granulocyte-derived EVs can increase the release of transforming growth factor-beta 1 (TGF-β1) to inhibit the macrophage inflammatory response to zymosan and LPS, indicating the essential anti-inflammatory role of these EVs [35].

LEVs are involved in coagulation and immune-inflammatory responses. These vesicles promote activation of the coagulation pathway via TF.T cell-derived EVs also positively regulate DC function, and B cell-derived extracellular vesicles impair the T cell response. Cell surface proteins (selectin, integrin, and complement regulators) on neutrophil-derived extracellular vesicles mediate the specific adherence of these extracellular vesicles to monocytes and endothelial cells. Therefore, LEVs induce inflammation-stimulating cellular signaling pathways. In addition, LEVs can increase TGF-β1 release from MDMs, which blocks the response of MDMs to LPS and zymosan.

LEVs are closely related to the immune response. Studies have shown that exosomes released from natural CD8^+^CD25^+^ regulatory T cells (Tregs) can inhibit CD8^+^ T cell responses and antitumor immunity [36]. In addition, miRNAs, especially miR-150-5p and miR-142-3p, are transferred from Tregs to dendritic cells (DCs) via Treg-derived EVs to inhibit immune reactions in tissues (with increased IL-10 and decreased IL-6 production after LPS stimulation) [37]. However, systemic immunosuppression severely reduces the antitumor effect of chemotherapy. EVs released from CD19^+^ B cells can impair the CD8^+^ T cell response. Therefore, inhibition of EVs derived from CD19^+^ B cells can improve the antitumor effect of chemotherapeutic agents [38]. In addition, LEVs can promote activation of the coagulation pathway via tissue factor (TF), which is the primary regulator of coagulation and hemostasis [39,40].

Monocyte-derived EVs may be involved in the immune response and inflammation in numerous diseases such as Alzheimer’s disease, multiple sclerosis, and stroke [41]. LEVs may be used as a biomarker for plaque vulnerability in patients with high-grade carotid stenosis [42]. MiRNAs, *e.g*., miR-146a, miR-128, miR-185, miR-365, and miR-503, contained in EVs released from atherogenic monocyte-derived macrophages (MDMs) may accelerate the development of atherosclerosis by decreasing cell migration and promoting macrophage entrapment in the vessel wall [43] (Table 1).

## 4. Extracellular vesicles derived from platelets

**Figure 4.**
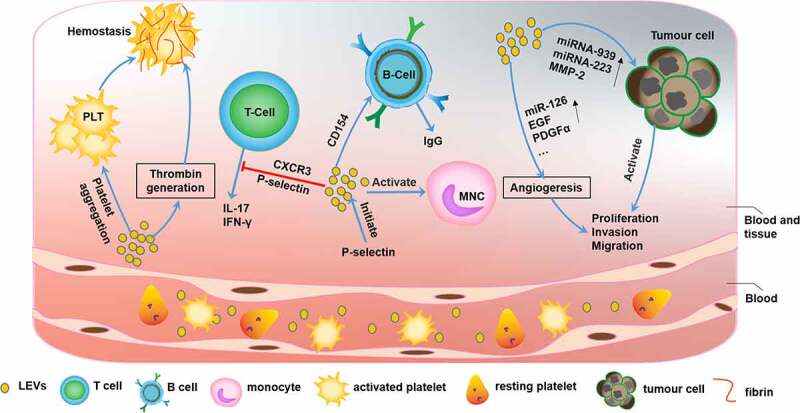
The function of platelet-derived extracellular vesicles (PEVs)

Platelets produced by megakaryocytes are involved mainly in physiological processes such as hemostasis and pathological processes such as thrombosis and inflammatory responses [44,45]. Similar to their parental cells, platelet-derived EVs (PEVs) are involved in various pathophysiological processes, including coagulation, infection, immune responses, angiogenesis, and tumorigenesis, via intracellular communication (Figure 4). The potential roles of PEVs in fibrinogenesis and resistance to fibrinolysis in hemostasis and thrombosis were demonstrated. When trauma occurs, PEV release is significantly increased, which promotes hemostasis and results in abundant thrombin generation, thus increasing platelet aggregation [46,47]. Conversely, PEVs may cause venous thrombosis [48]. In addition, PEVs are also associated with adverse blood transfusion reactions, such as urticaria, fever, erythema, dyspnea, and hypotension [49].

PEVs are involved in various biological processes, such as coagulation, immune-inflammatory responses, and tumorigenesis. PEVs can enhance platelet aggregation and thrombin formation. Additionally, PEVs also inhibit IL-17 and IFN-γ production by T cells via P-selectin and CXCR3 while stimulating the monocyte inflammatory response through P-selectin-dependent adhesion. In addition, PEVs also promote IgG secretion by delivering CD154 to B cells. During tumor progression, PEVs can increase tumor angiogenesis by increasing the content of miR-126, EGF, and PDGF-α. These vesicles directly promote tumor cell proliferation, invasion, and metastasis via elevated levels of miRNA-939, miRNA-223, and MMP-2.

It is becoming increasingly clear that platelets are intimately connected with infection and inflammation [50]. PEV formation is initiated by P-selectin-dependent adhesion to monocytes, and PEVs are stabilized by the binding of phosphatidylserine, which leades to an inflammatory response [51]. Similarly, PEVs have been shown to be closely associated with immune inflammation, which can inhibit IL-17 and IFN-γ production by Tregs in a P-selectin-dependent and partially CXCR3-dependent manner [52]. Moreover, PEVs can deliver CD154 to B cells and induce efficient IgG production [53]. Several studies have supported the role of PEVs in the development of chronic inflammation-related immune diseases, such as atherosclerosis [54], systemic lupus erythematosus [55], and renal inflammation [56]. A new study showed that platelet-derived serotonin promoted the transit of PEVs to lymph nodes, activated autoantibodies, increased vascular permeability, and promoted the progression of rheumatoid arthritis [57] (Table 1). As immune cells, platelets have an inherent affinity for inflammatory sites [44,58]. Qingle Ma et al. showed that PEVs accumulated in pneumonia sites and may be used as carriers of anti-inflammatory drugs to load pneumonia sites with [5-(p-fluorophenyl)-2-ureido] thiophene-3-carboxamide (TPCA-1), which inhibit the production of inflammatory factors and pulmonary infiltration to significantly improve the therapeutic effect [59] (Table 2).

PEVs are also related to tumor biology [60]. PEVs promote tumor proliferation, change the tumor microenvironment, and facilitate tumor metastasis. The contents of PEVs, such as miRNA-939, miRNA-223, and MMP-2, are key players in tumorigenesis [61]. Notably, PEVs affect angiogenesis [62] during cancer progression via the overexpression of miR-126 [63], epidermal growth factor (EGF), and platelet-derived growth factor-alpha (PDGFα) [64].

## 5. Extracellular vesicles derived from plasma

Peripheral blood plasma is a blood component that contains EVs derived from various cells, including erythrocytes, leukocytes, platelets, and other cells, e.g., tumor cells and endotheliocytes. Plasma EVs mediate hemostasis, inflammation, and injury responses [65,66]. Plasma EVs also play roles in disease diagnosis, prognosis, and therapy (Table 1). The protein and miRNA contents of exosomes isolated from the plasma of AML patients may be better than more commonly used biomarkers for diagnosing and predicting the recurrence of leukemia, and high levels of plasma EV-related TGF-β1 significantly inhibit natural killer (NK) cell activity to mediating disease recurrence [67]. In addition, plasma EVs may be used to monitor disease processes that occur in the cerebrum. MiR-212 and miR-132 are downregulated in neuron-derived plasma exosomes of Alzheimer’s disease patients [68]. Similarly, elevated expression levels of α-syn in plasma EVs activate microglia and astroglia, which enhancing neurodegeneration, the diagnostic marker of Parkinson’s disease [69,70].

Additionally, the potential of miRNAs in plasma EVs as novel diagnostic biomarkers for gastric cancer has been discussed extensively [71]. Other studies have shown that the significantly elevated levels of miR-21 and miR-1246 in plasma exosomes can indicate breast cancer occurrence [72]. In addition, the potential role of plasma EVs as a predictive tool in castrate-resistant prostate cancer (CRPC) diagnosis and during posttreatment follow-up has been reported: exosomal androgen receptor splice variant 7 (AR-V7) is associated with lower sex steroid levels and a shorter time to progression (median, 16.0 vs. 28.0 months; P = 0.0499) in CRPC patients [73]. Hoshino A et al [74]. detected the proteomics of plasma-derived extracellular vesicles and particles (EVPs) from healthy people and patients with different tumors. They found that the expression level of plasma-derived EVP proteins varied between tumors, which suggests that plasma-derived EVP proteins may be useful as liquid biopsy tests for cancer detection.

Plasma EVs have been used as delivery vehicles for therapeutic miRNAs and siRNAs. Plasma EVs engineered with antitumor miRNAs (miR-31 and miR-451a) promote the apoptosis of HepG2 liver cancer cells via the silencing of target genes in antiapoptotic pathways [75] (Table 2). Additionally, siRNAs may be loaded into plasma exosomes and delivered to monocytes and lymphocytes, leading to selective genetic silencing of mitogen-activated protein kinase-1 (MAPK-1) [76].

## 6. Targeted modification of blood cell-derived extracellular vesicles

Natural extracellular vesicles have low targeting ability, which seriously affects their application in the precise treatment of systemic diseases. Cargoes may be safely and efficiently delivered to specific cell types or tissues via targeted modifications of EVs [77,78]. Methods for targeted modification may be roughly categorized as genetic engineering modification and chemical modification methods [79]. Genetic engineering modifications are carried out by fusing the sequence of the gene encoding a target protein with the gene sequence encoding an EV membrane protein and transfecting the expression plasmid into parental cells. Then, EVs derived from the parental cells are modified.

**Figure 5.**
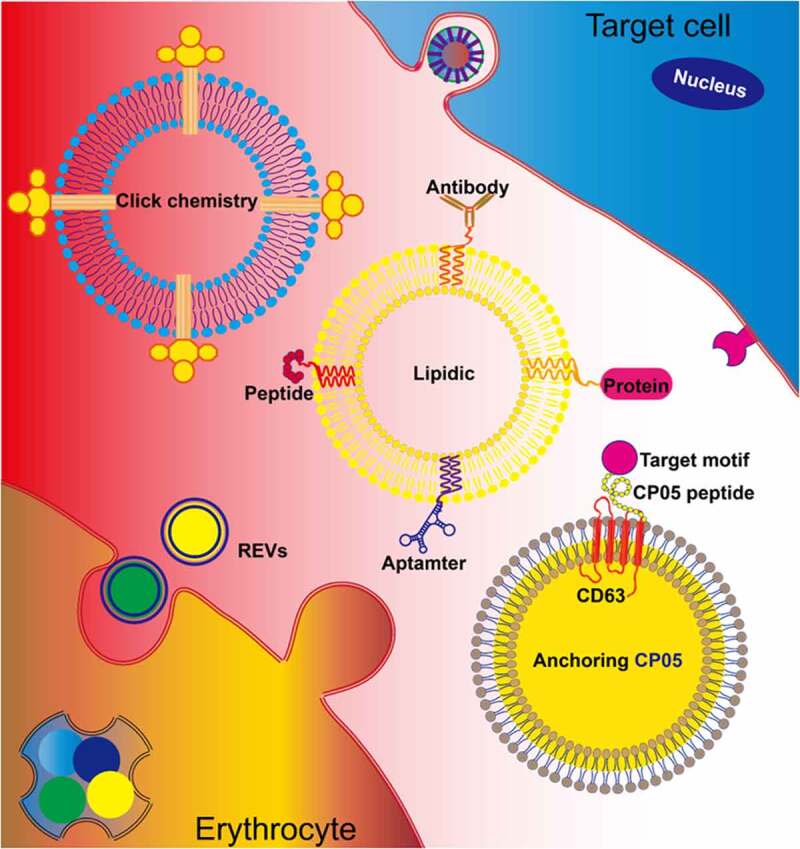
**Erythrocyte-derived extracellular vesicles** (**REVs) for therapeutic delivery** To optimize the targeted delivery of REVs, chemical modifications introduced by approaches such as click chemistry and lipidation can anchor the targeted motifs, including proteins, aptamers, peptides, and antibodies, on the exosomal membrane. Additionally, REVs can be modified using the CP05 peptide, with affinity for CD63, to introduce an exogenous target motif for display on the exosomal membrane

Extracellular vesicles derived from erythrocytes lack nuclear DNA and mitochondrial DNA and do not result in horizontal gene transfer [9]. Therefore, these vesicles may be excellent alternative therapeutic drug carriers. However, targeting strategies through genetic modification cannot be used with REVs, and alternative methods, such as chemical modification, could be developed. Currently, chemical modifications of EV membranes mainly involve peptides, antibodies, aptamers, small molecules via click chemistry [80], lipid-lipid interactions [81], or membrane-bound protein interactions [82] (Figure 5). At present, there is also a more optimized method for the targeted modification of EVs, which could bind EVs to the targeted peptide using through protein ligases, without changing the physicochemical properties of EV and with good stability and security. However, the method also has limitations, such as showing a lack of high affinity and specificity targeted nanobodies used [83]. In our previous study, we established a protocol for large-scale production of REVs from peripheral blood erythrocytes. Continuous efforts will be made to chemically modify REVs to recognize specific cells and improve the drug delivery efficiency for disease treatment.

## 7. Conclusions and perspectives

Extracellular vesicles derived from peripheral blood greatly participate in pathophysiological processes, such as coagulation, inflammation, immune responses, and tumor progression. Currently, extracellular vesicles derived from peripheral blood are extensively studied as potential biomarkers or disease predictors. More importantly, extracellular vesicles derived from erythrocytes show excellent prospects as particularly suitable sources for EV mimetics due to their unique characteristic of lacking nuclear material.

However, several crucial issues should be addressed before the clinical translation of blood cell-derived extracellular vesicles. Current precipitation strategies for large-scale EV production should be improved, since ultracentrifugation(UC) can destroy the EV nanostructure and induce their aggregation. Although UC is generally considered the ‘gold standard’ method for extracellular vesicle isolation, it may destroy the vesicle nanostructure and induce vesicle aggregation [84]. Currently, according to vesicle size, density, quality, and surface protein, studies have reported other methods for vesicle separation, such as polymer precipitation, which is highly specific but may be stained by other coprecipitated substances [85]. Ultrafiltration is simple and fast to perform, but filtration can change the morphology and influence downstream analysis [86]. The immunoaffinity capture method always shows specific binding, but its high cost may restrict widespread use [87]. The microfluidic-based method exhibits the advantages of strong sensitivity and high recovery, but it has clogging and size overlapping problems [88]. Each method has advantages and potential limitations, and no single method can be used for all types of samples. Therefore, it will be necessary to develop efficient and reliable EV separation and detection methods in the future. In addition, the high systemic clearance rate of extracellular vesicles before they reach target sites severely compromises the treatment outcome [89]. Therefore, the specific targeted modification of extracellular vesicles can improve the efficiency of drug delivery and therapeutic effects. With in-depth characterization of their biological behaviors and exploitation of novel modification strategies, extracellular vesicles derived from peripheral blood may be used to diagnose and treat more diseases.

## Data Availability

All data generated in the current study were included in this article.

## Highlights

Peripheral blood-derived extracellular vesicles participate in pathophysiological processes.

Peripheral blood-derived extracellular vesicles serve as alternative biomarkers for disease prediction.

Erythrocyte-derived extracellular vesicles serve as ideal drug carriers.

## Abbreviations

Antisense oligonucleotides (ASOs); androgen receptor splice variant 7 (AR-V7); B lymphocyte induced mature protein 1 (Blimp-1); blood-brain barrier (BBB); castrate-resistant prostate cancer (CRPC); dendritic cells (DCs); extracellular vesicles (EVs); extracellular vesicles and particles(EVPs); erythrocyte-derived EVs (REVs); excitatory amino acid transporter 2 (EAAT2); epidermal growth factor (EGF); factor XI (FXI); interferon regulatory factor 4 (IRF4); interleukin 1 (IL-1), interleukin 6 (IL-6);lipopolysaccharide (LPS); leukocyte-derived EVs (LEVs); mitogen-activated protein kinase-1 (MAPK-1); macrophage-derived chemokine (MDC); macrophage inflammatory protein 1a (MIP-1a); monocyte-derived macrophages (MDMs); nanoparticle tracking analysis (NTA); platelet-derived EVs (PEVs); platelet-derived growth factor-alpha (PDGFα);transfusion-related immune modulation(TRIM); tumor necrosis factor-α (TNF-α)),; transmission electron microscopy (TEM);transforming growth factor-beta 1 (TGF-β1); thiophene-3-carboxamide (TPCA-1); α-synuclein (α-syn).
